# Supplementary material for: The UFMylation pathway is impaired in Alzheimer’s disease
Source: Mol Neurodegener. 2024 Dec 18;19:97. doi: 10.1186/s13024-024-00784-y (PMC11656649; doi:10.1186/s13024-024-00784-y)
Supplement: Supplementary file 1 — Supplementary Material 1 [file 13024_2024_784_MOESM1_ESM.pdf]

SUPPLEMENTARY INFORMATION

The UFMylation pathway is impaired in Alzheimer's disease

Tingxiang Yan[1], Michael Heckman[2], Emily C. Craver[2], Chia-Chen Liu[1], Melissa E. Murray[1,3], Dennis W. Dickson[1,3], Xue Wang[1], Nilufer Ertekin-Taner[1,3], Zhenkun Lou[4], Guojun Bu[1,#], Wolfdieter Springer[1,3] and Fabienne C. Fiesel[1,3,*]

Affiliations:

[1] Department of Neuroscience, Mayo Clinic, Jacksonville, FL 32224, USA

[2] Division of Clinical Trials and Biostatistics, Mayo Clinic, Jacksonville, FL, USA

[3] Neuroscience PhD Program, Mayo Clinic Graduate School of Biomedical Sciences, Jacksonville, FL 32224, USA

[4]Department of Oncology, Mayo Clinic, Rochester, MN, 55905, USA

[#] present address: Division of Life Science, The Hong Kong University of Science and Technology, Hong Kong, China

* Corresponding author

Correspondence should be addressed to:

E-mail: Fiesel.Fabienne@mayo.edu

Keywords: UFMylation, UFM1, UFSP2, Alzheimer's disease, tau, brain

SUPPLEMENTARY FIGURE LEGENDS

Supplementary Figure 1: UFMylation gene expression is dysregulated in AD excitatory neurons

Meta-analysis of published gene expression data derived from single nuclei transcriptomics from AD patients versus controls [35]. (**A**) Heatmap of relative gene expression changes of all eight UFMylation pathway components in excitatory neurons (EX), inhibitory neurons (IN), astrocytes (AS), oligodendrocytes (OL), oligodendrocyte precursor cells (OPC), and microglia (MG). (**B**) Heatmap of relative gene expression changes of ubiquitin-like (UBL) pathways components in excitatory neurons. Significance levels used are as follows: *****: a gene meets 2-sided Wilcoxon-rank-sum test, FDR<0.01, |log-Fold-Change|>0.25, ******: a gene meets 2-sided Wilcoxon-rank-sum test, FDR<0.01, |log-Fold-Change|>0.25, and Poisson mixed-model FDR<0.05. See Supplementary Table S4 for related data.

Supplementary Figure 2: Optimization and assessments of the MSD ELISA for UFM1

(**A**) Western blot confirming the absence of UFM1 in UFM1 KO neurons, which were generated by CRISPR-Cas9. Black arrowhead indicates monomeric UFM1, gray arrowheads point to conjugated UFM1 species. Asterisks denote non-specific bands. (**B**) Comparison of five commercial UFM1 antibodies using WT, UFM1 KO and UFSP2 KO neuron samples for western blot, respectively. Ab1 (Abcam, ab109305) showed a clear difference between WT and UFM1 cells as well as between WT and UFSP2 cells. Ab2 (Sigma, HPA039758), Ab3 (Protein Tech Group, 15883-1-AP) and Ab4 (LS Bio, LS-C807041) show additional bands in UFSP2 KO cells, suggesting that they can detect the abundant conjugated form of UFM1 in these cells but do not show a clear difference between WT and UFM1 KO cells. Ab5 (LS Bio, LS-C500000) seems unspecific since there is no difference between WT, UFM1 KO and UFSP2 KO cells. (**C**) MSD ELISA test with the same five commercial UFM1 antibodies using denatured lysates from WT, UFM1 KO and UFSP2 KO neurons. Only Ab1 is suitable for MSD ELISA. Statistical significance was assessed with one-way ANOVA. Asterisks indicate statistical difference to WT (****P< 0.0001). (**D**) Lysates from UFM1 KO neurons were mixed with lysates from UFSP2 KO neuron to generate lysates with different concentrations of UFM1. Shown is a linear regression of UFM1 MSD ELISA values against this range of UFM1 concentrations.

Supplementary Figure 3: UFSP2 MSD ELISA

(**A**) Western blot of wild-type (-) and three different neuronal clones in which UFSP2 had been knocked out using CRISPR-Cas9. All UFSP2 KO neurons show lower level of unconjugated (black arrowhead) but higher levels of conjugated UFM1 (gray arrowheads). (**B**) MSD ELISA to measure UFSP2 using denatured lysates from WT, UFM1, or UFSP2 KO neurons. Statistical comparison to WT was assessed with one-way ANOVA followed by Dunnett's posthoc test: *P<0.05. (**C**) Spearman correlation of UFSP2 protein level in samples of the exploratory cohort (controls (blue) and AD (maroon)) measured by western blot and MSD ELISA. The correlation coefficient and P value are indicated. (**D**) Analysis of MSD ELISA-assessed soluble UFSP2 protein levels by disease group. Statistical analysis was performed by Wilcoxon rank sum test: **P<0.01. (**E, F**) Spearman correlation of MSD ELISA-assessed soluble UFSP2 protein levels with insoluble total UFM1 levels in samples of the exploratory cohort (controls (blue) and AD (maroon)) indicate a strong, significant negative correlation for both proteins in the frontal cortex (E) and the cerebellum (F). The correlation coefficient and P value are indicated. (**G**) Analysis of MSD ELISA-assessed UFM1 and UFSP2 protein levels in the soluble and insoluble fraction of cerebellum by disease group. Statistical analysis was performed by Wilcoxon rank sum test: ns, not significant.

Supplementary Figure 4: The insoluble fraction lacks deUFMylation activity

Brain tissue or RIPA-insoluble pellet was homogenized in reaction buffer and incubated with UFM1-GFP protein for 8 hours at 37°C. Cleavage of this fusion protein into its constituent parts, GFP and UFM1, is interpreted as peptidase activity. Negative and positive control samples were treated without brain homogenate (-) or with recombinant UFSP1 protein (+). Shown is a representative western blot with n=3 control and AD cases each to monitor cleavage activity with GFP and UFM1 antibodies. Grey arrowheads indicate uncleaved UFM1-GFP, while the cleaved forms that are detected with UFM1 or with GFP antibody are indicated by black arrowheads. Note that AD cases have comparable total UFSP2 level, but considerable higher UFSP2 levels in the insoluble fraction compared to controls, consistent with the results obtained in other experiments.

Supplementary Figure 5: Correlations of UFM1 and UFSP2 proteins with AD-related proteins that were biochemically measured from the temporal cortex

Heatmap of Spearman correlation coefficients (r_S) illustrating the absence of significant correlations of soluble and insoluble UFM1 and UFSP2 with most AD markers except for tau. Levels of ApoE, Aβ40, Aβ42, tau, and pT231-tau were previously measured in a separate study from the same individuals using three fractions: tris buffered saline (TBS), Triton X-100 (TX) and

formic acid (FA) [32]. A significant correlation of between soluble UFM1 and soluble total and pT231-tau in the TBS fraction, as well as insoluble UFM1 and pT231-tau in the TBS and TX fraction prompted us to investigate tau further. *P<0.05, **P<0.01.

Supplementary Figure 6: Knock out of UFSP2 does not affect the DNA damage response in undifferentiated neuronal precursor cells.

(**A**) Undifferentiated WT, UFM1 KO and UFSP2 KO neuronal precursors were treated with 10 µM etoposide for the indicated times and stained with an antibody against γH2AX. γH2AX intensity was measured by high content imaging. Intensity values were averaged and are presented as mean ± SEM from three independent experiments. Statistical significance was assessed with two-way ANOVA. No significant difference was identified. (**B**) Percentage of live neuronal precursor cells (WT, UFM1 KO, or UFSP2 KO) upon treatment with etoposide or bleomycin for 72 h. Cells were fixed and stained with viability/cytotoxicity dyes, imaged and analyzed by high content imaging. The number of live cells was normalized to the cell count of DMSO-treated cells for each genotype. Shown is the mean ± SEM of four independent experiments. Statistical significance was assessed with one-way ANOVA followed by Dunnett's post-hoc test: no significant difference was identified.

LIST OF SUPPLEMENTARY TABLES

Supplementary Table 1: Subject characteristics of exploratory cohort.

Supplementary Table 2: Subject characteristics of main cohort

Supplementary Table 3: Transformation of variables for linear regression analyses

Supplementary Table 4: Differential single nuclei expression of UBL pathway components in samples with and without AD pathology

Supplementary Table 5: Correlation of UFM1 values with other UFMylation proteins in exploratory cohort

Supplementary Table 6: Correlation of UFSP2 with UFM1 in human brain

Supplementary Table 7: Associations of primary clinical and disease parameters with UFSP2 and UFM1

Supplementary Table 8: Correlations of UFSP2 and UFM1 with common AD-related parameters in temporal cortex of AD patients

Supplementary Table 9: Comparison of tau measurements in between subject groups

Supplementary Table 10: Correlations of UFM1 and UFSP2 protein with expression levels of DNA damage related genes in the temporal cortex of AD patients

Supplementary Table 11: Correlation of UFM1 and UFSP2 with mRNA levels of unfolded protein response genes in the temporal cortex of AD patients

SFig 1: UFMylation gene expression is dysregulated in AD excitatory neurons

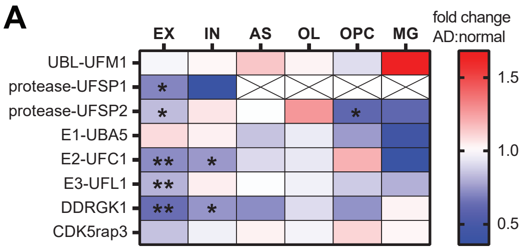

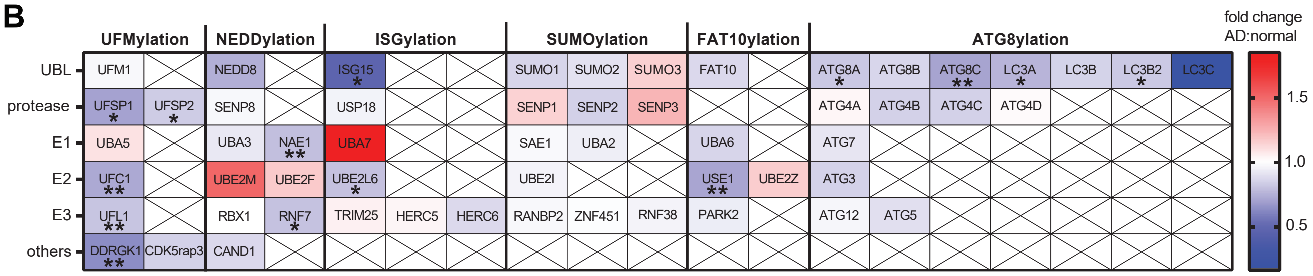

SFig 2: Optimization and assessments of the MSD ELISA for UFM1

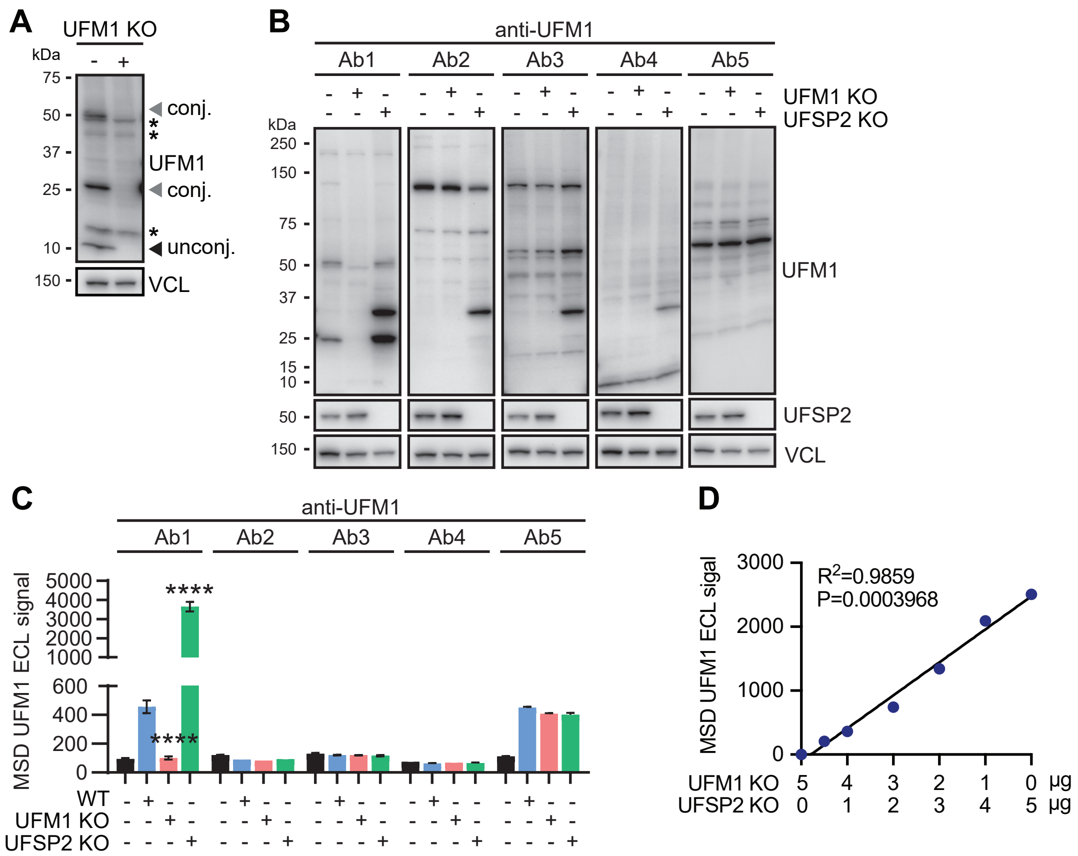

SFig 3: Assessments of MSD ELISA of UFSP2

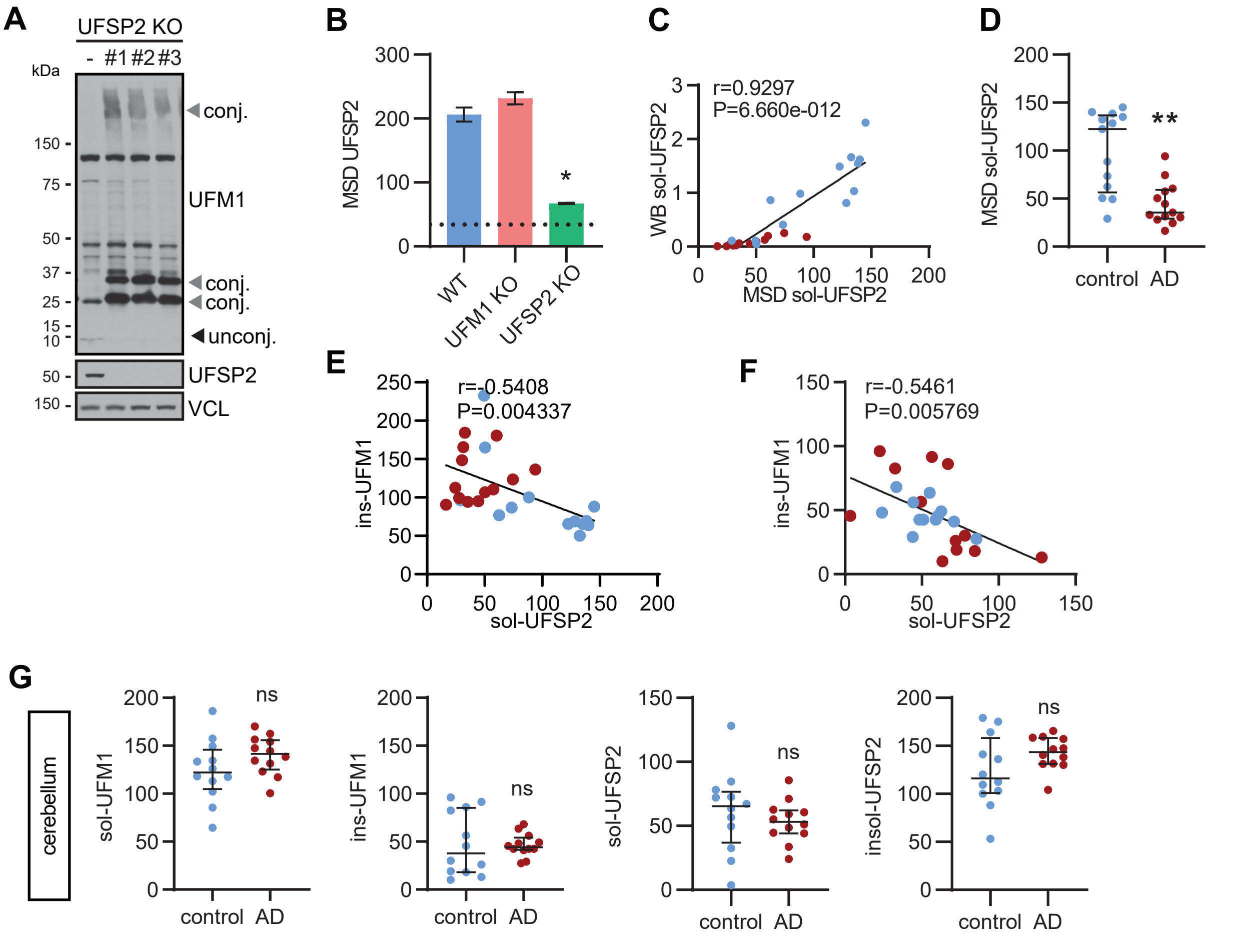

SFig 4: The insoluble fraction lacks deUFMylation activity

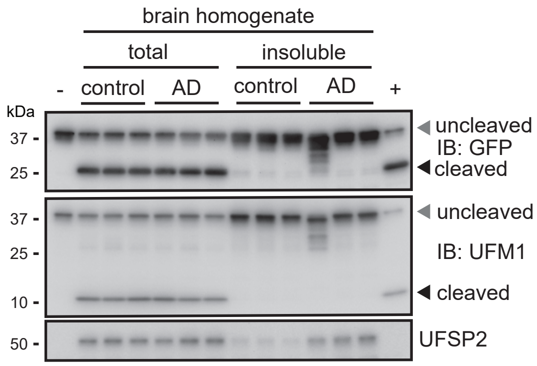

SFig 5: Correlations of UFM1 and UFSP2 with biochemical AD measures from the temporal cortex

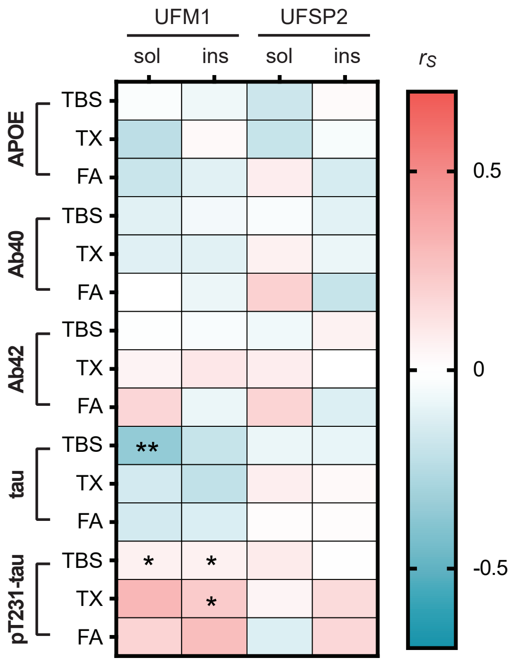

SFig 6: UFSP2 KO does not affect susceptibility of neuroprogenitor cells towards DNA damage

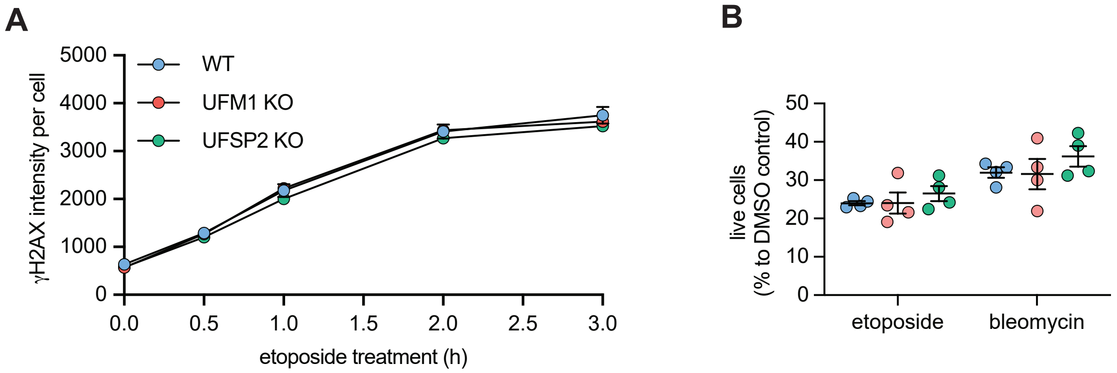

Supplemental Table 1: Subject characteristics of exploratory cohort.

**Cohort**	**N**	**Male** (%)	**age** median (min, max)	**disease duration** median (min, max)	**Braak tangle stage** median (min, max)	**Thal amyloid phase** median (min, max)
control	13	7 (53.8%)	73 (63, 91)	NA	0 (0, 3)	1 (0, 1)
AD	13	6 (46.2%)	80 (64, 89)	8 (7, 16)	6 (5, 6)	5 (5, 5)

AD=Alzheimer’s disease. Age and neuropathological scores are presented as median (min, max).

Supplemental Table 2: Subject characteristics

	AD patients (N=72)		Controls (N=41)		
Variable	N	Median (minimum, maximum) or No. (%) of patients	N	Median (minimum, maximum) or No. (%) of patients	P-value
Age (years)	72	82.3 (57.8, 95.9)	41	85.5 (70.5, 94.7)	0.1220
Sex (Male)	72	37 (51.4%)	41	22 (53.7%)	0.8469
APOE genotype	72		22		N/A
ε2/ε3		3 (4.2%)		1 (4.5%)	
ε2/ε4		2 (2.8%)		0 (0.0%)	
ε3/ε3		24 (33.3%)		17 (77.3%)	
ε3/ε4		32 (44.4%)		4 (18.2%)	
ε4/ε4		11 (15.3%)		0 (0.0%)	
Disease duration (years)	71	8.8 (2.4, 23.8)	0	N/A	N/A
MMSE score	61	13 (0, 27)	0	N/A	N/A
Braak stage	72		41		N/A
0		0 (0.0%)		1 (2.4%)	
I		0 (0.0%)		5 (12.2%)	
II		0 (0.0%)		8 (19.5%)	
III		0 (0.0%)		27 (65.9%)	
IV		17 (23.6%)		0 (0.0%)	
V		33 (45.8%)		0 (0.0%)	
VI		22 (30.6%)		0 (0.0%)	
Thal phase	72		41		N/A
0		0 (0.0%)		19 (46.3%)	
1		0 (0.0%)		4 (9.8%)	
2		0 (0.0%)		8 (19.5%)	
3		6 (8.3%)		10 (24.4%)	
4		9 (12.5%)		0 (0.0%)	
5		57 (79.2%)		0 (0.0%)	
Biochemical data (temporal cortex)	72				
apoE TBS		480.7 (7.8, 1244.4)		N/A	N/A
apoE TX		243.4 (33.8, 524.3)		N/A	N/A
apoE FA		330.8 (47.4, 1499.2)		N/A	N/A
Aβ40 TBS		69.8 (0.0, 8212.2)		N/A	N/A
Aβ40 TX		271.8 (8.2, 9482.8)		N/A	N/A
Aβ40 FA		964.7 (68.1, 186998.4)		N/A	N/A
Aβ42 TBS		631.2 (174.7, 2800.6)		N/A	N/A
Aβ42 TX		1511.6 (363.8, 6234.5)		N/A	N/A
Aβ42 FA		57.9 (12.8, 220.2)		N/A	N/A
tau TBS		4.7 (0.1, 10.6)		N/A	N/A
tau TX		1488.0 (63.1, 4134.1)		N/A	N/A
tau FA		11.7 (1.5, 27.1)		N/A	N/A
pT231-tau TBS		10.5 (1.9, 26.1)		N/A	N/A
pT231-tau TX		5.4 (1.3, 16.9)		N/A	N/A
pT231-tau FA		1404.9 (57.0, 11311.7)		N/A	N/A

AD=Alzheimer’s disease; MMSE=Mini mental state examination; TBS, TX, FA represent different sequential fractions. P-values result from a Wilcoxon rank sum test (continuous and ordinal variables) or Fisher’s exact test (categorical variables).

Supplemental Table 3: Transformation of variables for linear regression analyses

Variable	Transformation
Age	None
Disease duration	None
MMSE score	None
Temporal cortex	
sol total tau	None
ins total tau	Square root
sol p396/404-tau	Square root
ins p396/404-tau	Cube root
sol UFSP2	Square root
ins UFSP2	None
sol UFM1	None
ins UFM1	None
Frontal cortex	
sol total tau	None
ins total tau	Square root
sol p396/404-tau	Cube root
ins p396/404-tau	Square root
sol UFSP2	Square root
ins UFSP2	None
sol UFM1	None
ins UFM1	None

MMSE=mini mental state examination; sol=soluble; ins=insoluble.

Supplemental Table 4: Differential single nuclei expression of UBL pathway components in samples with and without AD pathology

			no-AD pathology vs AD pathology differential expression							
Cell type	Role of genes	Genes	IndModel .adj.pvals	no.pathology. mean	pathology. mean	FC	MixedModel. z	MixedModel. p	DEGs.In d.Model	DEGs.Ind.Mix. models
EX	UBL	UFM1	2.07E-26	0.1666	0.1620	0.9724	-0.4130	0.6796	FALSE	FALSE
IN	UBL	UFM1	1.26E-02	0.1057	0.1080	1.0220	-0.1872	0.8515	FALSE	FALSE
AS	UBL	UFM1	5.34E-01	0.0344	0.0394	1.1445	-0.7531	0.4514	FALSE	FALSE
OL	UBL	UFM1	6.46E-01	0.0409	0.0423	1.0347	0.3771	0.7061	FALSE	FALSE
OPC	UBL	UFM1	8.57E-01	0.0550	0.0504	0.9159	-0.3088	0.7574	FALSE	FALSE
MG	UBL	UFM1	7.60E-01	0.0196	0.0330	1.6818	0.6719	0.5016	FALSE	FALSE
EX	UFM1 Protease	UFSP1	7.18E-03	0.0031	0.0022	0.7053	NA	NA	**TRUE**	FALSE
IN	UFM1 Protease	UFSP1	1.85E-01	0.0014	0.0005	0.3602	NA	NA	FALSE	FALSE
AS	UFM1 Protease	UFSP1	N/A							
OL	UFM1 Protease	UFSP1	N/A							
OPC	UFM1 Protease	UFSP1	N/A							
MG	UFM1 Protease	UFSP1	N/A							
EX	UFM1 Protease	UFSP2	3.56E-16	0.0669	0.0559	0.8347	-0.0223	0.9822	**TRUE**	**FALSE**
IN	UFM1 Protease	UFSP2	2.24E-01	0.0615	0.0655	1.0654	0.0434	0.9654	FALSE	FALSE
AS	UFM1 Protease	UFSP2	9.49E-01	0.0492	0.0489	0.9938	0.7409	0.4587	FALSE	FALSE
OL	UFM1 Protease	UFSP2	7.69E-03	0.0354	0.0450	1.2706	2.7633	0.0057	**TRUE**	FALSE
OPC	UFM1 Protease	UFSP2	6.15E-01	0.0186	0.0113	0.6112	-1.6260	0.1039	FALSE	FALSE
MG	UFM1 Protease	UFSP2	6.15E-01	0.0186	0.0113	0.6112	-1.6260	0.1039	FALSE	FALSE
EX	UFM1 E1	UBA5	1.76E-20	0.1002	0.1095	1.0930	1.4667	0.1424	FALSE	FALSE
IN	UFM1 E1	UBA5	7.18E-02	0.0565	0.0588	1.0413	-0.1776	0.8591	FALSE	FALSE
AS	UFM1 E1	UBA5	2.65E-01	0.0529	0.0452	0.8542	-1.6192	0.1054	FALSE	FALSE
OL	UFM1 E1	UBA5	9.36E-01	0.0150	0.0142	0.9483	-0.2372	0.8125	FALSE	FALSE
OPC	UFM1 E1	UBA5	5.74E-01	0.0566	0.0429	0.7584	-1.3782	0.1681	FALSE	FALSE
MG	UFM1 E1	UBA5	9.39E-01	0.0121	0.0053	0.4386	-2.2390	0.0252	FALSE	FALSE
EX	UFM1 E2	UFC1	1.63E-75	0.1422	0.1026	0.7220	-5.5931	0.0000	**TRUE**	**TRUE**
IN	UFM1 E2	UFC1	3.06E-06	0.0618	0.0465	0.7525	-2.4667	0.0136	**TRUE**	FALSE
AS	UFM1 E2	UFC1	6.49E-01	0.0521	0.0472	0.9057	-0.1046	0.9167	FALSE	FALSE
OL	UFM1 E2	UFC1	8.94E-01	0.0314	0.0295	0.9413	0.4133	0.6794	FALSE	FALSE
OPC	UFM1 E2	UFC1	9.29E-01	0.0437	0.0526	1.2021	0.8315	0.4057	FALSE	FALSE
MG	UFM1 E2	UFC1	1.66E-01	0.0358	0.0131	0.3665	-2.6684	0.0076	FALSE	FALSE
EX	UFM1 E3	UFL1	3.59E-57	0.1418	0.1156	0.8155	-4.7652	0.0000	**TRUE**	**TRUE**
IN	UFM1 E3	UFL1	7.45E-03	0.1058	0.1107	1.0464	-0.0663	0.9471	FALSE	FALSE

AS	UFM1 E3	UFL1	5.50E-01	0.1507	0.1494	0.9914	-0.1384	0.8899	FALSE	FALSE
OL	UFM1 E3	UFL1	4.22E-01	0.0957	0.0918	0.9590	-1.1643	0.2443	FALSE	FALSE
OPC	UFM1 E3	UFL1	6.15E-01	0.1531	0.1331	0.8697	-0.5380	0.5906	FALSE	FALSE
MG	UFM1 E3	UFL1	9.68E-01	0.0439	0.0355	0.8081	-0.4989	0.6179	FALSE	FALSE
EX	UFM1 E3 complex	DDRGK1	3.40E-140	0.2460	0.1580	0.6422	-6.8151	0.0000	**TRUE**	**TRUE**
IN	UFM1 E3 complex	DDRGK1	1.87E-07	0.0768	0.0571	0.7431	-1.0690	0.2851	**TRUE**	FALSE
AS	UFM1 E3 complex	DDRGK1	6.18E-02	0.0930	0.0675	0.7256	-0.8582	0.3908	FALSE	FALSE
OL	UFM1 E3 complex	DDRGK1	6.66E-01	0.0619	0.0566	0.9146	-1.5869	0.1125	FALSE	FALSE
OPC	UFM1 E3 complex	DDRGK1	7.25E-01	0.0707	0.0522	0.7385	-0.4086	0.6829	FALSE	FALSE
MG	UFM1 E3 complex	DDRGK1	9.11E-01	0.0125	0.0129	1.0332	0.2798	0.7797	FALSE	FALSE
EX	UFM1 E3 complex	CDK5RAP3	2.87E-57	0.1903	0.1619	0.8508	-1.1861	0.2356	FALSE	FALSE
IN	UFM1 E3 complex	CDK5RAP3	4.18E-03	0.1078	0.1031	0.9566	0.5762	0.5645	FALSE	FALSE
AS	UFM1 E3 complex	CDK5RAP3	7.78E-01	0.1971	0.2048	1.0393	0.2635	0.7922	FALSE	FALSE
OL	UFM1 E3 complex	CDK5RAP3	8.25E-01	0.1124	0.1085	0.9649	-0.6207	0.5348	FALSE	FALSE
OPC	UFM1 E3 complex	CDK5RAP3	9.54E-01	0.1393	0.1545	1.1096	1.5649	0.1176	FALSE	FALSE
MG	UFM1 E3 complex	CDK5RAP3	8.81E-01	0.0812	0.0833	1.0251	0.3773	0.7060	FALSE	FALSE
EX	UBL	ISG15	6.95E-50	0.0531	0.0253	0.4759	-3.3039	0.0010	**TRUE**	FALSE
EX	UBL	NEDD8	1.71E-01	0.0007	0.0005	0.7471	NA	NA	FALSE	FALSE
EX	UBL	SUMO1	6.66E-71	0.3539	0.3060	0.8647	-2.4086	0.0160	FALSE	FALSE
EX	UBL	SUMO2	2.11E-22	0.0626	0.0566	0.9033	-1.8748	0.0608	FALSE	FALSE
EX	UBL	SUMO3	3.66E-15	0.2843	0.3279	1.1534	3.3783	0.0007	FALSE	FALSE
EX	UBL	FAT10	2.06E-91	0.5297	0.4623	0.8728	-3.0750	0.0021	FALSE	FALSE
EX	UBL (ATG8)	GABARAP	1.91E-06	0.0287	0.0235	0.8218	0.2695	0.7876	**TRUE**	FALSE
EX	UBL (ATG8)	GABARAPL1	3.53E-90	0.5040	0.4369	0.8670	-3.5538	0.0004	FALSE	FALSE
EX	UBL (ATG8)	GABARAPL2	1.79E-145	0.5547	0.3918	0.7063	-4.9819	0.0000	**TRUE**	**TRUE**
EX	UBL (ATG8)	MAP1LC3A	1.48E-100	0.3627	0.2764	0.7622	-3.2186	0.0013	**TRUE**	FALSE
EX	UBL (ATG8)	MAP1LC3B	5.98E-68	0.3353	0.2876	0.8577	-4.7490	0.0000	FALSE	FALSE
EX	UBL (ATG8)	MAP1LC3B2	6.48E-23	0.0440	0.0362	0.8234	-3.9275	0.0001	**TRUE**	FALSE
EX	NEDD8 Protease	SENP8	3.63E-08	0.0314	0.0304	0.9679	0.8687	0.3850	FALSE	FALSE
EX	ISG15 Protease	USP18	4.44E-07	0.0137	0.0131	0.9536	-0.8783	0.3798	FALSE	FALSE
EX	SUMO Protease	SENP1	1.11E-08	0.0632	0.0723	1.1444	2.9842	0.0028	FALSE	FALSE
EX	SUMO Protease	SENP2	5.56E-69	0.2796	0.2380	0.8514	-1.7946	0.0727	FALSE	FALSE
EX	SUMO Protease	SENP3	8.36E-01	0.0002	0.0002	1.2371	NA	NA	FALSE	FALSE
EX	SUMO Protease	SENP5	3.59E-66	0.3714	0.3432	0.9240	-1.6980	0.0895	FALSE	FALSE
EX	SUMO Protease	SENP6	1.30E-49	0.5918	0.5936	1.0031	1.9740	0.0484	FALSE	FALSE
EX	SUMO Protease	SENP7	3.60E-38	0.2050	0.1996	0.9734	-0.2334	0.8155	FALSE	FALSE
EX	ATG8 Protease	ATG4A	8.58E-06	0.0256	0.0265	1.0318	0.4072	0.6839	FALSE	FALSE
EX	ATG8 Protease	ATG4B	1.62E-105	0.3164	0.2696	0.8521	-2.2549	0.0241	FALSE	FALSE
EX	ATG8 Protease	ATG4C	5.83E-64	0.1667	0.1420	0.8520	-4.4933	0.0000	FALSE	FALSE

EX	ATG8 Protease	ATG4D	4.51E-22	0.0818	0.0833	1.0189	-0.5735	0.5663	FALSE	FALSE
EX	ISG15 E1	UBA7	3.69E-01	0.0009	0.0017	1.8508	NA	NA	FALSE	FALSE
EX	NEDD8 E1	UBA3	9.15E-56	0.2511	0.2318	0.9230	-0.6925	0.4886	FALSE	FALSE
EX	NEDD8 E1	NAE1	7.10E-90	0.2427	0.1933	0.7967	-4.7057	0.0000	**TRUE**	**TRUE**
EX	SUMO E1	SAE1	3.52E-43	0.2413	0.2350	0.9741	-0.0079	0.9937	FALSE	FALSE
EX	SUMO E1	UBA2	8.91E-62	0.3508	0.3298	0.9400	-0.3340	0.7383	FALSE	FALSE
EX	ATG8 E1	ATG7	5.75E-102	0.8162	0.7454	0.9132	-4.6332	0.0000	FALSE	FALSE
EX	ISG15 E2	UBE2L6	1.88E-21	0.0510	0.0409	0.8018	-2.5585	0.0105	**TRUE**	FALSE
EX	NEDD8 E2	UBE2M	7.81E-02	0.2491	0.3755	1.5076	3.3878	0.0007	FALSE	FALSE
EX	NEDD8 E2	UBE2F-SCLY	1.38E-06	0.0245	0.0287	1.1719	0.3064	0.7593	FALSE	FALSE
EX	FAT10 E2	USE1	3.30E-44	0.0841	0.0600	0.7141	-4.9904	0.0000	**TRUE**	**TRUE**
EX	FAT10 E2	UBE2Z	1.64E-21	0.2347	0.2725	1.1610	2.5947	0.0095	FALSE	FALSE
EX	ATG12 E2	ATG10	5.47E-104	0.2630	0.2049	0.7790	-2.5290	0.0114	**TRUE**	FALSE
EX	SUMO E2	UBE2I	2.85E-44	0.2413	0.2305	0.9552	0.1179	0.9061	FALSE	FALSE
EX	ATG8 E2	ATG3	2.30E-17	0.0641	0.0548	0.8547	-0.8928	0.3719	FALSE	FALSE
EX	SUMO E3	RNF38	1.78E-47	0.3094	0.2962	0.9572	0.0675	0.9461	FALSE	FALSE
EX	ISG15 E3	TRIM25	8.11E-04	0.0328	0.0345	1.0510	2.0130	0.0441	FALSE	FALSE
EX	ISG15 E3	HERC5	4.75E-01	0.0025	0.0026	1.0354	NA	NA	FALSE	FALSE
EX	NEDD8 E3	RBX1	4.81E-50	0.4145	0.4160	1.0036	0.5073	0.6119	FALSE	FALSE
EX	NEDD8 E3	RNF7	2.29E-46	0.1320	0.1065	0.8068	-3.2946	0.0010	**TRUE**	FALSE
EX	SUMO E3	RANBP2	4.20E-65	0.4989	0.4845	0.9711	-1.4443	0.1487	FALSE	FALSE
EX	SUMO E3	ZNF451	1.37E-52	0.4200	0.4146	0.9872	0.8150	0.4151	FALSE	FALSE
EX	SUMO E3	RNF38	1.78E-47	0.3094	0.2962	0.9572	0.0675	0.9461	FALSE	FALSE
EX	FAT10 E3	PARK2	2.47E-75	1.1431	1.0713	0.9372	-3.0734	0.0021	FALSE	FALSE
EX	ATG8 E3	ATG12	4.48E-37	0.2013	0.1935	0.9609	-0.1696	0.8653	FALSE	FALSE
EX	ATG8 E3	ATG5	2.34E-62	0.2100	0.1885	0.8976	-4.9821	0.0000	FALSE	FALSE

Meta analysis of published single cell transcriptome data [35]. EX=excitatory neurons, IN=inhibitory neurons, AS=astrocytes, OL=oligodendrocytes, OPC=oligodendrocyte precursor cells, MG=microglia, UBL=ubiquitin-like protein. IndModel.adj.pvals= fdr-adjusted p-values, two-sided 2-sided Wilcoxon-rank-sum test, FC=fold change of AD pathology mean value relative to no-AD pathology mean value, MixedModel.z=z-score of Poisson mixed model, MixedModel.p=P-value of Poisson mixed model, DEGs.Ind.Model=logical indication of whether a gene meets the criteria fdr-adjusted P-value<0.01 and absolute log2 fold change > 0.25, DEGs.Ind.Mix.models=logical indication of whether a gene meets the criteria fdr-adjusted MixedModel.P<0.05 (frd-adjustment over genes meeting criteria in DEGs.Ind.Model)

Supplemental Table 5: Correlation of UFM1 values with other UFMylation proteins in exploratory cohort

	**r_s (P-value)**							
Variable	free UFM1	UFSP1	UFSP2	UBA5	UFC1	UFL1	DDRGK1	CDK5RAP3
total sol UFM1	0.14<br>(0.4901)	0.01<br>(0.9709)	**-0.56**<br>**(0.0028)**	-0.33<br>(0.0956)	0.14<br>(0.485)	-0.43<br>(0.028)	-0.12<br>(0.5435)	-0.36<br>(0.0668)
total ins UFM1	-0.31<br>(0.121)	-0.24<br>(0.228)	**-0.66**<br>**(0.0002)**	-0.48<br>(0.0142)	0.22<br>(0.2699)	-0.27<br>(0.1808)	0.03<br>(0.8827)	-0.41<br>(0.0395)

rs=Spearman correlation coefficient; sol=soluble, ins=insoluble. Total UFM1 was determined by MSD ELISA in the RIPA-soluble and -insoluble fraction in frontal cortex tissue of the exploratory cohort (all subjects combined). Other variables were determined by western blot of the soluble fraction. P-values < 0.0055 were considered statistically significant after applying a Bonferroni correction for multiple testing. Significant associations are shown in bold.

Supplemental Table 6: Correlation of UFSP2 with UFM1 in human brain

	Association with sol UFSP2				Association with ins UFSP2			
	unadjusted analysis		multivariable analysis		unadjusted analysis		multivariable analysis	
Cortex/Group/Variable	β (95% CI)	P-value	β (95% CI)	P-value	β (95% CI)	P-value	β (95% CI)	P-value
**Temporal Cortex**								
Controls (N=41)								
sol UFSP2	N/A	N/A	N/A	N/A	-0.71 (-1.60, 0.18)	0.1128	-0.76 (-1.71, 0.18)	0.1108
ins UFSP2	-0.71 (-1.60, 0.18)	0.1128	-0.76 (-1.71, 0.18)	0.1108	N/A	N/A	N/A	N/A
sol UFM1	-0.01 (-1.03, 1.01)	0.9808	-0.03 (-1.10, 1.05)	0.9603	9.79 (-2.93, 22.50)	0.1276	8.60 (-4.74, 21.93)	0.1993
ins UFM1	-0.93 (-1.49, -0.36)	0.0019	**-0.94 (-1.59, -0.29)**	**0.0058**	2.27 (-5.89, 10.42)	0.5776	4.47 (-4.59, 13.52)	0.3238
AD patients (N=72)								
sol UFSP2	N/A	N/A	N/A	N/A	-1.03 (-1.46, -0.59)	<0.0001	**-1.01 (-1.46, -0.57)**	**<0.0001**
ins UFSP2	-1.03 (-1.46, -0.59)	<0.0001	**-1.01 (-1.46, -0.57)**	**<0.0001**	N/A	N/A	N/A	N/A
sol UFM1	0.24 (-0.24, 0.72)	0.3304	0.19 (-0.31, 0.69)	0.4435	11.73 (3.78, 19.69)	0.0044	**12.96 (4.69, 21.23)**	**0.0026**
ins UFM1	-1.25 (-1.81, -0.69)	<0.0001	**-1.24 (-1.84, -0.64)**	**0.0001**	16.56 (6.28, 26.83)	0.0020	**18.45 (7.52, 29.37)**	**0.0013**
All subjects (N=113)								
sol UFSP2	N/A	N/A	N/A	N/A	-0.96 (-1.35, -0.58)	<0.0001	**-0.96 (-1.36, -0.57)**	**<0.0001**
ins UFSP2	-0.96 (-1.35, -0.58)	<0.0001	**-0.96 (-1.36, -0.57)**	**<0.0001**	N/A	N/A	N/A	N/A
sol UFM1	0.19 (-0.24, 0.62)	0.3822	0.15 (-0.29, 0.60)	0.4995	11.17 (4.57, 17.77)	0.0009	**11.72 (4.86, 18.59)**	**0.0008**
ins UFM1	-1.09 (-1.47, -0.70)	<0.0001	**-1.10 (-1.53, -0.67)**	**<0.0001**	9.05 (-4.94, 23.04)	0.2047	11.10 (-2.59, 24.78)	0.1119
**Frontal Cortex**								
Controls (N=41)								
sol UFSP2	N/A	N/A	N/A	N/A	-1.37 (-2.23, -0.52)	0.0024	**-1.85 (-2.83, -0.86)**	**0.0005**
ins UFSP2	-1.37 (-2.23, -0.52)	0.0024	**-1.85 (-2.83, -0.86)**	**0.0005**	N/A	N/A	N/A	N/A
sol UFM1	-1.03 (-2.36, 0.30)	0.1266	-1.13 (-2.58, 0.32)	0.1213	26.93 (8.04, 45.83)	0.0064	19.89 (1.23, 38.54)	0.0373
ins UFM1	-1.73 (-2.54, -0.93)	<0.0001	**-1.95 (-2.83, -1.06)**	**<0.0001**	9.38 (-5.20, 23.96)	0.2007	12.02 (-2.11, 26.14)	0.0929
AD patients (N=72)								
sol UFSP2	N/A	N/A	N/A	N/A	-1.46 (-2.18, -0.74)	0.0001	**-1.46 (-2.24, -0.69)**	**0.0003**
ins UFSP2	-1.46 (-2.18, -0.74)	0.0001	**-1.46 (-2.24, -0.69)**	**0.0003**	N/A	N/A	N/A	N/A
sol UFM1	-0.61 (-1.28, 0.06)	0.0734	-0.62 (-1.34, 0.10)	0.0892	12.01 (3.24, 20.79)	0.0080	**11.45 (2.24, 20.67)**	**0.0156**
ins UFM1	-1.47 (-2.25, -0.69)	0.0003	**-1.51 (-2.37, -0.65)**	**0.0008**	18.71 (8.17, 29.26)	0.0007	**17.96 (6.55, 29.36)**	**0.0025**
All subjects (N=113)								
sol UFSP2	N/A	N/A	N/A	N/A	-1.42 (-1.96, -0.89)	<0.0001	**-1.61 (-2.21, -1.02)**	**<0.0001**
ins UFSP2	-1.42 (-1.96, -0.89)	<0.0001	**-1.61 (-2.21, -1.02)**	**<0.0001**	N/A	N/A	N/A	N/A
sol UFM1	-0.70 (-1.28, -0.11)	0.0199	-0.73 (-1.36, -0.10)	0.0240	17.20 (3.27, 31.12)	0.0155	**13.15 (5.07, 21.23)**	**0.0014**
ins UFM1	-1.60 (-2.14, -1.05)	<0.0001	**-1.73 (-2.33, -1.13)**	**<0.0001**	15.33 (6.55, 24.12)	0.0006	**15.57 (6.92, 24.22)**	**0.0004**

sol=soluble; ins=insoluble; β=regression coefficient; CI=confidence intervals; AD=Alzheimer's disease. β values, 95% CIs, and P-values for the separate control and AD groups result from linear regression models. β values are interpreted as the increase in mean UFSP2 (on the square root scale when examining sol UFSP2) corresponding to 1 SD increase for the shown continuous variables, which were examined untransformed, or square root transformed (sol UFSP2). Multivariable models for controls were adjusted for age, sex, Braak stage, and Thal phase, and multivariable models for AD patients were adjusted for age, sex, presence of *APOE* ε4, Braak stage, and Thal phase. β values, 95% CIs, and P-values for the analysis of all subjects results from a random effects meta-analysis combining the separate results from the control and AD groups. After applying a Bonferroni correction for multiple testing P-values < 0.0167 are considered as statistically significant. Significant findings from the multivariate analysis are shown in bold. Note: there was no significant association of soluble UFM1 with insoluble UFM1 in any of the models.

Supplemental Table 7: Associations of primary clinical and disease parameters with UFSP2 and UFM1

	Association with sol UFSP2		Association with ins UFSP2		Association with sol UFM1		Association with ins UFM1	
Cortex/Group/Variable	β (95% CI)	P-value	β (95% CI)	P-value	β (95% CI)	P-value	β (95% CI)	P-value
**Temporal Cortex**								
Controls (N=41)								
Age (10 year increase)	0.66 (-0.57, 1.88)	0.2841	5.34 (-10.23, 20.90)	0.4913	4.17 (-26.98, 35.32)	0.7875	-30.26 (-55.19, -5.32)	0.0188
Sex (Male)	-0.20 (-1.91, 1.52)	0.8186	12.72 (-9.06, 34.50)	0.2439	11.10 (-32.49, 54.69)	0.6088	-11.15 (-46.05, 23.74)	0.5209
Presence of *APOE* ε4	-0.05 (-3.49, 3.38)	0.9742	-15.26 (-55.46, 24.93)	0.4353	-18.92 (-107.04, 69.20)	0.6573	9.35 (-17.80, 36.50)	0.4787
Braak stage (1 unit increase)	0.19 (-0.80, 1.17)	0.7014	-0.36 (-12.89, 12.16)	0.9535	8.66 (-16.42, 33.73)	0.4883	-6.68 (-26.75, 13.39)	0.5041
Thal phase (1 unit increase)	-0.07 (-0.69, 0.56)	0.8328	4.82 (-3.14, 12.77)	0.2274	5.04 (-10.89, 20.96)	0.5252	-5.04 (-17.78, 7.71)	0.4282
AD patients (N=72)								
Age (10 year increase)	0.50 (-0.25, 1.25)	0.1892	-4.82 (-18.03, 8.39)	0.4690	21.35 (-8.03, 50.74)	0.1515	-6.10 (-17.95, 5.75)	0.3079
Sex (Male)	-0.31 (-1.39, 0.76)	0.5619	6.83 (-12.14, 25.80)	0.4748	-15.74 (-57.94, 26.47)	0.4592	-7.04 (-24.06, 9.98)	0.4121
Presence of *APOE* ε4	-0.22 (-1.31, 0.86)	0.6834	3.77 (-15.40, 22.94)	0.6961	7.63 (-35.25, 50.51)	0.7236	4.60 (-12.90, 22.09)	0.6016
Disease duration (5 year increase)	0.58 (-0.15, 1.31)	0.1164	-2.26 (-15.42, 10.91)	0.7332	-7.03 (-36.25, 22.18)	0.6322	-9.38 (-20.88, 2.11)	0.1077
MMSE score (5 unit increase)	0.08 (-0.44, 0.60)	0.7553	1.89 (-7.28, 11.06)	0.6810	3.22 (-16.50, 22.94)	0.7446	4.19 (-3.61, 11.99)	0.2864
Braak stage (1 unit increase)	-0.22 (-1.02, 0.59)	0.5899	-4.09 (-18.26, 10.07)	0.5659	5.13 (-26.37, 36.62)	0.7464	9.54 (-3.18, 22.25)	0.1392
Thal phase (1 unit increase)	-0.06 (-0.98, 0.87)	0.9059	3.21 (-13.15, 19.56)	0.6966	15.45 (-20.92, 51.82)	0.3996	1.22 (-13.46, 15.91)	0.8684
All subjects (N=113)								
Age (10 year increase)	0.54 (-0.08, 1.17)	0.0890	-0.49 (-10.33, 9.36)	0.9229	13.13 (-7.70, 33.96)	0.2166	-15.78 (-38.98, 7.42)	0.1826
Sex (Male)	-0.28 (-1.17, 0.61)	0.5380	9.42 (-4.53, 23.36)	0.1857	-2.54 (-32.08, 27.00)	0.8660	-7.85 (-22.82, 7.12)	0.3042
Presence of *APOE* ε4	-0.21 (-1.22, 0.81)	0.6900	-0.06 (-16.89, 16.76)	0.9940	2.11 (-35.37, 39.60)	0.9121	6.10 (-8.12, 20.32)	0.4008
Braak stage (1 unit increase)	-0.05 (-0.66, 0.56)	0.8646	-1.97 (-11.10, 7.16)	0.6723	7.31 (-11.76, 26.39)	0.4524	3.20 (-12.31, 18.70)	0.6861
Thal phase (1 unit increase)	-0.06 (-0.57, 0.44)	0.8082	4.52 (-2.42, 11.45)	0.2017	6.67 (-7.46, 20.80)	0.3551	-2.39 (-11.76, 6.97)	0.6162
**Frontal Cortex**								
Controls (N=41)								
Age (10 year increase)	0.69 (-1.06, 2.43)	0.4321	12.72 (-10.47, 35.92)	0.2732	8.29 (-15.09, 31.67)	0.4768	-9.93 (-37.86, 18.00)	0.4754
Sex (Male)	0.02 (-2.43, 2.46)	0.9889	14.29 (-18.16, 46.74)	0.3777	12.63 (-20.08, 45.34)	0.4387	0.76 (-38.32, 39.83)	0.9688
Presence of *APOE* ε4	0.13 (-3.96, 4.23)	0.9472	19.31 (-33.42, 72.03)	0.4518	6.69 (-48.69, 62.07)	0.8025	40.17 (-16.96, 97.29)	0.1569
Braak stage (1 unit increase)	-0.09 (-1.50, 1.32)	0.8970	**25.32 (6.66, 43.99)**	**0.0092**	11.71 (-7.10, 30.53)	0.2149	-11.07 (-33.54, 11.41)	0.3246
Thal phase (1 unit increase)	-0.04 (-0.93, 0.86)	0.9358	7.37 (-4.48, 19.23)	0.2152	2.05 (-9.90, 14.00)	0.7300	14.58 (0.31, 28.86)	0.0455
AD patients (N=72)								
Age (10 year increase)	21.35 (-8.03, 50.74)	0.1515	-6.10 (-17.95, 5.75)	0.3079	-17.09 (-37.06, 2.87)	0.0920	-12.51 (-26.42, 1.40)	0.0772
Sex (Male)	-15.74 (-57.94, 26.47)	0.4592	-7.04 (-24.06, 9.98)	0.4121	-19.04 (-47.72, 9.63)	0.1894	-5.46 (-25.44, 14.52)	0.5870
Presence of *APOE* ε4	7.63 (-35.25, 50.51)	0.7236	4.60 (-12.90, 22.09)	0.6016	22.43 (-6.72, 51.57)	0.1293	8.99 (-11.76, 29.74)	0.3905
Disease duration (5 year increase)	-7.03 (-36.25, 22.18)	0.6322	-9.38 (-20.88, 2.11)	0.1077	-3.89 (-23.74, 15.95)	0.6964	3.62 (-10.21, 17.44)	0.6029
MMSE score (5 unit increase)	3.22 (-16.50, 22.94)	0.7446	4.19 (-3.61, 11.99)	0.2864	-3.06 (-14.44, 8.32)	0.5923	7.60 (-1.61, 16.82)	0.1039
Braak stage (1 unit increase)	5.13 (-26.37, 36.62)	0.7464	9.54 (-3.18, 22.25)	0.1392	-10.63 (-32.39, 11.12)	0.3329	3.07 (-11.90, 18.04)	0.6833
Thal phase (1 unit increase)	15.45 (-20.92, 51.82)	0.3996	1.22 (-13.46, 15.91)	0.8684	9.55 (-15.57, 34.68)	0.4504	15.45 (-1.83, 32.74)	0.0788
All subjects (N=113)								
Age (10 year increase)	13.13 (-7.70, 33.96)	0.2166	-15.78 (-38.98, 7.42)	0.1826	-5.05 (-29.89, 19.79)	0.6902	-11.98 (-24.17, 0.20)	0.0539
Sex (Male)	-2.54 (-32.08, 27.00)	0.8660	-7.85 (-22.82, 7.12)	0.3042	-4.06 (-35.05, 26.94)	0.7975	-4.14 (-21.55, 13.27)	0.6410
Presence of *APOE* ε4	2.11 (-35.37, 39.60)	0.9121	6.10 (-8.12, 20.32)	0.4008	18.73 (-6.31, 43.77)	0.1426	14.46 (-8.78, 37.69)	0.2227
Braak stage (1 unit increase)	7.31 (-11.76, 26.39)	0.4524	3.20 (-12.31, 18.70)	0.6861	1.27 (-20.58, 23.12)	0.9091	-1.64 (-14.71, 11.42)	0.8052
Thal phase (1 unit increase)	6.67 (-7.46, 20.80)	0.3551	-2.39 (-11.76, 6.97)	0.6162	3.40 (-7.06, 13.86)	0.5242	**14.93 (4.22, 25.63)**	**0.0063**

sol=soluble; ins=insoluble; β=regression coefficient; CI=confidence intervals; AD=Alzheimer's disease. β values, 95% CIs, and P-values for the separate control and AD groups result from multivariate linear regression models. β values are interpreted as the increase in mean UFSP2 (on the square root scale when examining sol UFSP2) corresponding to

presence of the given characteristic (categorical variables) or the increase given in parenthesis (continuous variables, which were examined on the untransformed, square root, cube root, or natural logarithm scale). Models for controls were adjusted for age, sex, Braak stage, and Thal phase, and models for AD patients were adjusted for age, sex, presence of *APOE* ε4, Braak stage, and Thal phase. β values, 95% CIs, and P-values for the analysis of all subjects results from a random effects meta-analysis combining the separate results from the control and AD groups. P-value < 0.01 (controls and all subjects) and < 0.0071 (AD patients) is considered as significant after applying a Bonferroni correction for multiple testing separately for each disease group and each outcome. Significant findings are shown in bold.

Supplemental Table 8: Correlations of UFSP2 and UFM1 with common AD-related parameters in temporal cortex of AD patients

	r_s (P-value)			
Variable Fraction	sol UFSP2	ins UFSP2	sol UFM1	ins UFM1
apoE TBS	-0.18 (0.1304)	0.028 (0.8174)	-0.023 (0.8486)	-0.061 (0.6115)
apoE TX	-0.194 (0.1024)	-0.036 (0.7655)	-0.223 (0.0601)	0.034 (0.7759)
apoE FA	0.085 (0.4771)	-0.14 (0.2419)	-0.186 (0.1178)	-0.102 (0.3936)
Ab40 TBS	-0.029 (0.8108)	-0.099 (0.4081)	-0.102 (0.3929)	-0.049 (0.6799)
Ab40 TX	0.07 (0.5573)	-0.074 (0.534)	-0.109 (0.3629)	-0.107 (0.372)
Ab40 FA	0.202 (0.0889)	-0.193 (0.1042)	-0.003 (0.9822)	-0.073 (0.5402)
Ab42 TBS	-0.059 (0.6245)	0.069 (0.5642)	-0.01 (0.9355)	-0.031 (0.7946)
Ab42 TX	0.085 (0.4753)	-0.007 (0.9559)	0.062 (0.6036)	0.109 (0.3631)
Ab42 FA	0.193 (0.1038)	-0.117 (0.3274)	0.18 (0.1297)	-0.074 (0.535)
tau TBS	-0.078 (0.5166)	-0.087 (0.4658)	**-0.345 (0.003)**	-0.194 (0.1028)
tau TX	0.083 (0.4869)	0.036 (0.7611)	-0.148 (0.2163)	-0.21 (0.0772)
tau FA	0.019 (0.874)	0.019 (0.8741)	-0.148 (0.2153)	-0.126 (0.2914)
pT231-tau TBS	0.098 (0.4107)	0.013 (0.9162)	0.072 (0.5465)	0.072 (0.546)
pT231-tau TX	0.058 (0.6293)	0.164 (0.1684)	**0.312 (0.0076)**	**0.232 (0.0496)**
pT231-tau FA	-0.121 (0.313)	0.177 (0.1379)	0.192 (0.107)	**0.289 (0.0138)**

rs= Spearman correlation coefficient; sol=soluble; ins=insoluble. TBS, TX and FA represent different sequential fractions. P-values < 0.05 were considered statistically significant in this exploratory analysis. Significant correlations are shown in bold.

Supplemental Table 9: Comparison of tau measurements in between subject groups

	AD patients (N=72)		Controls (N=41)		
Variable	N	Median (minimum, maximum) or No. (%) of patients	N	Median (minimum, maximum) or No. (%) of patients	P-value
Temporal cortex					
sol total tau	72	107743.8 (40783.6, 227510.3)	41	113608.5 (27590.5, 212176.9)	0.7974
ins total tau	72	38463.0 (2575.7, 155918.1)	41	4929.2 (941.5, 15080.3)	**<0.0001**
sol pS396/404-tau	72	830.8 (70.4, 7527.1)	41	144.3 (0.0, 521.3)	**<0.0001**
Ins pS396/404-tau	72	68363.6 (196.4, 199058.8)	41	624.3 (0.0, 22494.3)	**<0.0001**
Frontal cortex					
sol total tau	72	102324.1 (35991.2, 168711.0)	41	95239.2 (22891.2, 156687.5)	0.1177
ins total tau	72	45697.8 (1970.9, 124103.5)	41	3538.5 (508.2, 7544.2)	**<0.0001**
sol pS396/404-tau	72	334.5 (0.0, 7220.5)	41	78.5 (6.5, 274.5)	**<0.0001**
ins pS396/404-tau	72	63434.7 (405.8, 182778.5)	41	246.0 (0.0, 7338.5)	**<0.0001**

AD=Alzheimer's disease; sol=soluble; ins=insoluble. P-values result from a Wilcoxon rank sum test. P-values P<0.0125 considered statistically significant after applying a Bonferroni correction separately for each cortex. Significant associations are highlighted in bold.

Supplemental Table 10: UFM1 and UFSP2 protein correlations with DDR gene expression in AD patient temporal cortex

	r_s (P-value)			
DNA damage response gene	sol UFSP2	ins UFSP2	sol UFM1	ins UFM1
TP53BP1	**0.4427 (0.0003)**	-0.1349 (0.2919)	-0.0556 (0.6652)	**-0.2723 (0.0308)**
XRCC4	-0.0088 (0.9454)	-0.0168 (0.896)	0.2182 (0.0858)	0.2089 (0.1004)
XRCC5	**0.3611 (0.0036)**	-0.2241 (0.0775)	0.1113 (0.3853)	-0.1476 (0.2483)
XRCC6	**0.6963 (<0.0001)**	-0.2271 (0.0735)	0.1958 (0.1242)	**-0.3413 (0.0062)**
PRKDC	0.1358 (0.2886)	-0.1792 (0.16)	-0.0382 (0.7664)	0.0029 (0.9819)
LIG4	**0.2484 (-0.0496)**	-0.0724 (0.5726)	0.061 (0.6346)	-0.2008 (0.1146)
DCLRE1C	**-0.5209 (<0.0001)**	0.1549 (0.2254)	0.0356 (0.7818)	**0.3793 (0.0022)**
NHEJ1	**-0.4578 (-0.0002)**	-0.0203 (0.8743)	-0.1612 (0.2069)	0.2408 (0.0573)
RAD50	0.0406 (0.7518)	-0.0469 (0.7152)	-0.1975 (0.1207)	-0.0274 (0.8312)
MRE11A	-0.1941 (0.1274)	0.0913 (0.4769)	0.017 (0.8945)	0.19 (0.1359)
NBN	0.2096 (0.0992)	0.0495 (0.6999)	**0.2795 (0.0265)**	0.0218 (0.8651)
ATR	**0.5763 (<0.0001)**	**-0.3692 (0.0029)**	-0.0969 (0.45)	**-0.3643 (0.0033)**
RPA1	0.0605 (0.6377)	0.0188 (0.8839)	0.2146 (0.0912)	0.1928 (0.1301)
RPA2	**0.6216 (<0.0001)**	**-0.3274 (0.0088)**	-0.059 (0.646)	**-0.2874 (0.0224)**
RPA3	**0.5648 (<0.0001)**	-0.2412 (0.0568)	0.0403 (0.754)	**-0.3803 (0.0021)**
RAD52	**-0.4823 (<0.0001)**	0.0441 (0.7316)	**-0.2529 (0.0455)**	**0.2562 (0.0427)**
BRCA1	**-0.4733 (<0.0001)**	0.0874 (0.4958)	-0.1402 (0.2733)	**0.2819 (0.0252)**
BRCA2	**-0.3655 (0.0032)**	-0.1035 (0.4194)	**-0.2735 (0.0301)**	0.2264 (0.0744)
RAD51	**-0.3311 (0.008)**	-0.0526 (0.6823)	-0.1921 (0.1314)	**0.2903 (0.021)**
PALB2	-0.0689 (0.5916)	-0.0609 (0.6353)	0.0129 (0.9201)	0.0246 (0.8484)
CHEK1	**-0.4362 (0.0004)**	0.0658 (0.6085)	-0.2144 (0.0915)	0.2 (0.116)
ATM	**-0.6344 (<0.0001)**	0.1036 (0.4192)	-0.1807 (0.1564)	**0.4192 (0.0006)**
CHEK2	**-0.4996 (<0.0001)**	0.0999 (0.436)	-0.0818 (0.5238)	**0.3096 (0.0135)**
TP53	**-0.305 (0.0151)**	0.0879 (0.4935)	0.1123 (0.3811)	**0.2659 (0.0352)**
WEE1	**-0.3486 (0.0051)**	**0.2603 (0.0393)**	**0.2598 (0.0398)**	**0.3429 (0.0059)**
MDM2	**-0.5028 (<0.0001)**	0.0817 (0.5243)	-0.0732 (0.5685)	**0.3693 (0.0029)**
CDKN1A	**-0.3514 (-0.0047)**	0.2199 (0.0834)	0.0345 (0.7884)	0.23 (0.0697)
GADD45A	0.2332 (0.0659)	0.0599 (0.6411)	0.0396 (0.7581)	-0.2018 (0.1127)
GADD45B	-0.0573 (0.6556)	0.1422 (0.2661)	0.0759 (0.5543)	0.0263 (0.8378)
GADD45G	**-0.3806 (0.0021)**	0.2275 (0.073)	0.1538 (0.2289)	0.237 (0.0614)
CDK1	**-0.2935 (0.0196)**	-0.0273 (0.8321)	-0.1468 (0.2509)	0.0893 (0.4866)
CDK2	**-0.3861 (0.0018)**	0.1382 (0.28)	0.1911 (0.1336)	**0.32 (0.0106)**

rs=Spearman correlation coefficient; sol=soluble; ins=insoluble. P-values < 0.05 are considered statistically significant. Significant correlations are shown in bold. DDR: DNA damage response.

Supplemental Table 11: UFM1 and UFSP2 protein correlations with seven UPR genes expression in AD patient temporal cortex

	r_s (P-value)			
unfolded protein response gene	sol UFSP2	ins UFSP2	sol UFM1	ins UFM1
HSPA5/Bip	-0.1358 (0.2886)	0.0436 (0.7345)	0.0255 (0.8427)	0.0911 (0.4776)
EIF2AK3/PERK	**-0.3025 (0.016)**	0.1514 (0.2363)	-0.048 (0.7088)	0.2215 (0.081)
ATF4	**-0.5855 (<0.0001)**	**0.2511 (0.0471)**	-0.0812 (0.5269)	**0.3133 (0.0124)**
DDIT3/CHOP	**-0.3001 (0.0169)**	0.0284 (0.8249)	-0.1445 (0.2584)	0.1391 (0.2769)
ERN1/IRE1α	**-0.2489 (0.0492)**	0.066 (0.6075)	0.0803 (0.5314)	**0.2606 (0.0391)**
XBP1	-0.1614 (0.2063)	0.1596 (0.2116)	0.0664 (0.605)	0.1689 (0.1858)
ATF6	**0.2696 (0.0326)**	-0.0294 (0.8189)	0.2133 (0.0932)	-0.0156 (0.9033)

rs=Spearman correlation coefficient; sol=soluble; ins=insoluble. P-values < 0.05 are considered statistically significant. Significant correlations are shown in bold. UPR: unfolded protein response.
